# Viability of *Lactobacillus reuteri* DSM 17938 Encapsulated by Ionic Gelation during Refractance Window^®^ Drying of a Strawberry Snack

**DOI:** 10.3390/foods13060823

**Published:** 2024-03-07

**Authors:** Esmeralda Mosquera-Vivas, Alfredo Ayala-Aponte, Liliana Serna-Cock, Cristian Torres-León, Diego F. Tirado

**Affiliations:** 1 Escuela de Ingeniería de Alimentos, Universidad del Valle, Cali 760031, Valle del Cauca, Colombia; esmeralda.mosquera@correounivalle.edu.co (E.M.-V.); alfredo.ayala@correounivalle.edu.co (A.A.-A.); 2 Facultad de Ingeniería y Administración, Universidad Nacional de Colombia, Sede Palmira, Palmira 763533, Valle del Cauca, Colombia; lserna@unal.edu.co; 3 Reaserch Center and Ethnobiological Garden, Universidad Autonoma de Coahuila, Viesca 27480, Coahuila, Mexico; ctorresleon@uadec.edu.mx; 4 Dirección Académica, Universidad Nacional de Colombia, Sede de La Paz, La Paz 202017, Cesar, Colombia

**Keywords:** probiotic, polymeric coating, alginate, pectin, whey protein concentrate, chitosan

## Abstract

The selection of appropriate probiotic strains is vital for their successful inclusion in foods. These strains must withstand processing to reach consumers with ≥10^6^ CFU/g, ensuring effective probiotic function. Achieving this in commercial products is challenging due to sensitivity to temperature during processing. In this work, *Lactobacillus reuteri* DSM 17938 was microencapsulated by ionic gelation (with alginate or pectin) followed by polymeric coating (with whey protein concentrate or chitosan). Then, such microcapsules were incorporated into a strawberry puree, which was subsequently dehydrated at three temperatures (40 °C, 45 °C, and 50 °C) by Refractance Window^®^. The ultimate aim was to demonstrate the efficacy of the proposed methods from a technological point of view. Kinetic curves of the probiotic’s viability showed a high cell loading (>10^9^ CFU/g). Additionally, an average encapsulation efficiency of 91% and a particle size of roughly 200 µm were found. A decrease in the viability of the microorganism was observed as drying temperature and time increased. As a demonstration of the above, in a particular case, drying at 45 °C and 50 °C, viable cells were found up to 165 min and 90 min, respectively; meanwhile, drying at 40 °C, viable cells were reported even after 240 min. The greatest viability preservation was achieved with Refractance Window^®^ drying at 40 °C for 240 min when microcapsules coated with whey protein concentrate were incorporated into puree; this procedure showed great potential to produce dehydrated strawberry snacks with moisture (15%), water activity (*a_w_* < 0.6), and viability (≥10^6^ CFU/g) suitable for functional foods. The membrane-stabilizing properties of whey protein concentrate could prevent cell damage. In contrast, probiotics in chitosan-coated capsules showed reduced viability, potentially due to antimicrobial properties and the formation of cracks. These findings signify a breakthrough in the production of dehydrated snacks with the addition of probiotics, addressing challenges in preserving the viability of these probiotics during processing; thus, opening the possibility for the development of a probiotic strawberry snack.

## 1. Introduction

In recent decades, the perception of food has evolved from its primary function as a source of nutrients and energy to its crucial role in promoting health and wellness. In this regard, probiotics, which are live microorganisms, have become a key component in the design of functional foods [1].

The choice of suitable probiotic strains for incorporation into foods is essential. These strains must be resistant to processing to ensure that they reach the intake stage and provide the consumer with a sufficient number of cells (≥10^6^ CFU/g) to perform their probiotic function. However, achieving this recommended level of probiotic bacteria in commercial products is challenging, as probiotics are extremely sensitive to high temperatures, a condition they face during processing [2].

Probiotics have been mainly added to liquid food matrices [3,4], as their inclusion in solid matrices poses challenges due to the need to ensure viability throughout the drying process. Thus, the design and development of these dehydrated solid foods require strategies to protect microorganisms through appropriate encapsulation and drying strategies that guarantee the viability of the probiotics in the final product [5].

Encapsulation is a widely used technique to improve the survival of probiotics during their incorporation into foods. Depending on the polymer used and the encapsulation technique, microcapsules can act as selectively permeable physical barriers that regulate the interaction between the probiotic and the environment. Several methods have been used to obtain capsules containing probiotics, the most common being spray drying, freeze drying, fluidized beds, double emulsions, complex coacervation, and ionic gelation [6].

Ionic gelation involves the use of hydrocolloids such as alginate, carrageenan, pectin, chitosan, and gelatin, among others [7], along with a solidifying solution such as calcium chloride. This technique can help reduce the loss of probiotic viability in fruit matrices subjected to adverse conditions during processing [8]. In the scientific literature, several investigations have shown promising results in the encapsulation of probiotic microorganisms using ionic gelation [8,9]. However, despite the benefits of ionic gelation, microcapsules often exhibit some degree of porosity, which can be problematic for the protection of probiotic bacteria [10]. To address this challenge, a coating polymer can be added to improve the stability of microcapsules obtained by ionic gelation [11].

A snack is a portion of food consumed between main meals, usually small or medium in size and easy to consume. These food products are prized for their convenience and versatility, making them a popular choice to satisfy hunger and cravings throughout the day. In this research, strawberry puree was used for the incorporation of a probiotic microorganism. Strawberry, appreciated worldwide for its aroma, color, and flavor, offers an ideal structure for the incorporation of probiotics and facilitates the drying process, allowing a snack to be obtained [12].

In addition to the above, drying is an energy-intensive unit operation, accounting for up to 20% of the total energy consumed in the food industry. Over 85% of industrial food dryers are convective, with mostly hot air as the media for heat transfer, often resulting in significant levels of changes in product quality from the initial fresh-like form [13]. In most cases, conventional drying methods produce inferior-quality products and require relatively long drying times. Therefore, there is a strong need for the development of alternative drying technologies, considering operational capacity, process control, time requirements, cost economics, product quality, safety, and environmental aspects [14,15]. In this regard, Refractance Window^®^ drying is an emerging drying technique with a potential positive impact on food engineering in terms of scalability, energy efficiency, cost, and end-product quality. Furthermore, this fourth-generation drying technique is especially suitable for dehydrating heat-sensitive foods, preserving their organoleptic and nutritional properties [15].

Based on the above, this research focused on demonstrating the efficacy, from a technological point of view, of preserving the survival of *Lactobacillus reuteri* DSM 17938 microencapsulated by ionic gelation (with alginate or pectin) followed by polymeric coating (with whey protein concentrate or chitosan) and added to a strawberry puree that was then dried by the Refractance Window^®^ process.

## 2. Materials and Methods

### 2.1. Materials and Reagents

Acetic acid (glacial, ≥99%, Sigma-Aldrich, Bogotá, Colombia); CaCl_2_ (beads, Sigma-Aldrich, Bogotá, Colombia); chitosan (low molecular weight, Sigma-Aldrich, Bogotá, Colombia); citric acid (≥99.5%, crystals, Sigma-Aldrich, Bogotá, Colombia); Man, Rogosa, and Sharpe broth (MRS, Sigma-Aldrich, Bogotá, Colombia); pectin (low methoxyl, Tecnas, Medellín, Colombia); peptone water (buffered, Scharlau, Barcelona, Spain); phosphate-buffered saline solution (PBS; 8 g/L NaCl, 0.2 g/L KCl, 1.44 g/L Na_2_HPO_4_, and 0.24 g/L KH_2_PO_4_, Sigma-Aldrich, Bogotá, Colombia); sodium alginate (Sigma-Aldrich, Bogotá, Colombia); sodium citrate (99.7%, Sigma-Aldrich, Bogotá, Colombia); sodium hydroxide (NaOH, ≥98%, pellets, anhydrous, Sigma-Aldrich, Bogotá, Colombia); and whey protein concentrate (ST plus 93X, Tecnas, Medellín, Colombia) were used for this study.

### 2.2. Probiotic Bacteria: Reproduction

*L. reuteri* DSM 17938 cells were obtained from the commercial product Reuterin™ (BioGaia^®^, Stockholm, Sweden) and cultured in MRS broth at 37 °C for 24 h (INE 400, Memmert, Schwabach, Germany). The strain was subcultured twice and maintained in MRS broth with 1% *v/v* inoculum for activation and adaptation.

Fermentations were carried out in a 500 mL Erlenmeyer flask with a working volume of 300 mL MRS substrate and 10% *v*/*v* inoculum at 37 °C for 24 h with orbital shaking (VWR^®^ 5000I Incubating Orbital Shaker, Visalia, CA, USA). The concentration of viable cells was determined by serial decimal dilutions in 0.1% *w/v* peptone water. Samples were plated on MRS agar plates and incubated at 37 °C for 48 h under aerobic conditions.

### 2.3. Microencapsulation of Lactobacillus reuteri DSM 1793 by Ion Gelation

Cells for encapsulation were collected from 300 mL of fermented material by centrifugation at 5000 rpm for 10 min (centrifuge 5804R, Eppendorf, Hamburg, Germany). Then, the pellet was washed with sterile PBS solution at pH 7.2.

Microencapsulation of *L. reuteri* in (i) alginate or (ii) pectin was performed as follows: cells obtained from the fermentation substrate were suspended and homogenized in (i) 100 mL of 2% *w*/*v* alginate solution or (ii) 100 mL of 2% pectin solution (common unsalted butter was added at 1% *w*/*v*). In both cases, the cell solution was added by atomization in 250 mL of 3% *w*/*v* CaCl_2_ solution using a conventional airbrush (Discover LN1, UTIL, Bogotá, Colombia) equipped with a 1.5 mm nozzle. After atomization, cells were allowed to stand for 15 min for crosslinking. Subsequently, cells microencapsulated in alginate or pectin were collected by filtration and washed with 250 mL of PBS solution. Nonencapsulated free *L. reuteri* cells (FC) were used as a control treatment for microencapsulation.

Alginate was used because of its degradability and biocompatibility and because it does not accumulate in biological systems [16]. Similarly, low-methoxyl pectin was used because of its ability to form gels under the process conditions, since neither sugar nor acid was required for gelation [17]. Finally, CaCl_2_ was used to favor the formation of polyvalent ionic bonds of both alginate and pectin. These bonds acted as crosslinking agents that resulted in the encapsulating matrix [18,19].

### 2.4. Polymeric Coating

After obtaining the alginate or pectin microcapsules, a polymeric coating was applied using (i) chitosan or (ii) whey protein concentrate. The alginate or pectin microcapsules were separately suspended in (i) 0.7% *w*/*v* chitosan solution in 0.1 M acetic acid at pH 3.0 or (ii) 4% *w*/*v* whey protein concentrate solution in distilled water at pH 4.0. Both suspensions were kept under magnetic stirring at 2400 rpm for 15 min (Corning, New York, NY, USA). Alginate or pectin microcapsules coated with chitosan were named A-C and P-C, respectively; while alginate or pectin microcapsules coated with whey protein concentrate were named A-WPC or P-WPC, respectively.

### 2.5. Z-Potential Analysis

The Z-potential of each solution (*i.e.*, alginate, pectin, whey protein concentrate, and chitosan) was measured by using a Malvern Zetasizer instrument (Zetasizer Nano S, Worcestershire, UK) at a sweep of pH 2–8, adjusted by addition of NaOH 3 M to raise the pH and citric acid 0.1 N to lower the pH of the solutions; in this way, a picture of the possible interaction window between the polymer solutions was obtained [20]. The pH value of the chitosan and whey protein concentrate solutions for the coating was chosen based on the Z-potential results, considering the interaction between positive and negative charges.

### 2.6. Viability of Free and Encapsulated Bacteria

The enumeration of *L. reuteri* DSM 17938 was performed on MRS agar. Microcapsules were disintegrated by adding 0.2 M sodium citrate solution at pH 7.0 followed by shaking at 2400 rpm for 5 min (Vortex Heidolph, Schwabach, Germany). Subsequently, serial dilutions were carried out in 0.1% *w/v* peptone water with incubation for 72 h. Sodium citrate was used to degrade the alginate or pectin network and promote cell release [21].

### 2.7. Encapsulation Efficiency

The encapsulation efficiency (% *EE*) of probiotics was calculated according to Equation (1), where *N* (CFU/g) was the number of viable cells released upon degradation of microparticles and *N*_0_ (CFU/g) was the number of viable cells in the cell concentrate before microencapsulation.

(1) $$\%\;EE=\left(\frac{N}{N_{0}}\right)\times 100\%$$

### 2.8. Capsule Size

The diameter of capsules was determined using an optical microscope with a calibrated micrometer (Nikon Eclipse e200, TV Lends 0.55X DS, Tokyo, Japan). About 20 capsules from each test were analyzed immediately after the encapsulation process. For this, samples were diluted in distilled water adjusted to the coating pH (*i.e*., pH 4.0 for whey protein concentrate and pH 3.0 for chitosan). A 0.1 mL volume of capsules was mixed in 5 mL of water through manual stirring in such a way that microscopic observation was possible independently.

### 2.9. Preparation of the Fruit Matrix

Strawberries (*Fragaria ananassa*) with a maturity stage between 5 and 6 according to Colombian Technical Standard (NTC, by its Spanish acronym) 4103 were used because strawberries at this maturity stage were homogeneous in color and had the highest total soluble solid content [22]. Strawberries were washed with potable water and immersed in distilled water with 5% acetic acid (1 part acetic acid/10 parts water) for 5 min. They were then cut into small pieces with a stainless steel knife and processed in a blender (Philips HR 1764, São Paulo, Brazil) until a puree was formed.

The strawberry puree was characterized in terms of moisture content (A.O.A.C. 930.15) with a drying oven (E 28 BINDER^®^, BINDER GmbH, Tuttlingen, Germany), soluble solids (A.O.A.C. 932. 12) with a refractometer (Atago, Model RX-7000α, Bellevue, WA, USA), and pH (A.O.A.C. 945.10) with a pH meter (Orion 710A, Cambridge, MA, USA) based on A.O.A.C. methods [23]. Finally, the water activity (*a_w_*) was determined using a water activity meter (AquaLab Series 3TE, Decagon Devices, Inc., Pullman, WA, USA).

### 2.10. Incorporation of Capsules into the Strawberry Puree

*L. reuteri* DSM 17938 cells, both in their free form (FC) and microencapsulated (A-C, A-WPC, P-C or P-WPC), were added to the strawberry puree in a sufficient quantity to reach a concentration of viable microorganisms of up to 10^9^ CFU/g. Each type of capsule was incorporated separately, with a composition of 20% *w*/*w* capsules and 80% *w/w* puree. In the case of free cells (FC), 20% *w*/*w* corresponded to cell pellet obtained from 300 mL of fermentation substrate.

### 2.11. Refractance Window^®^ Drying

**Figure 1:**
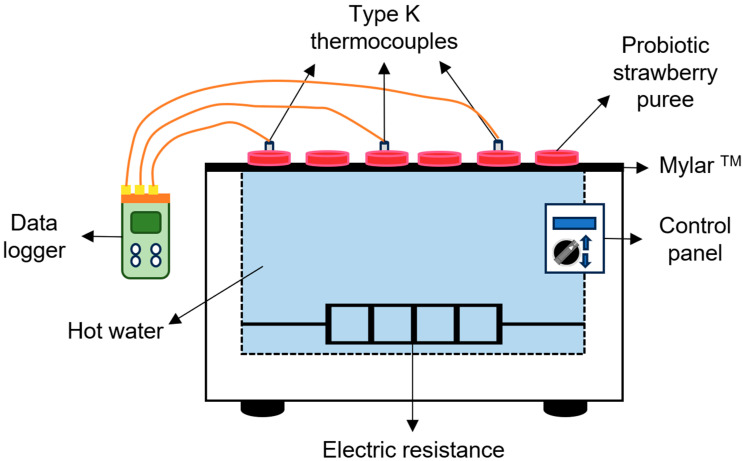
Diagram of the Refractance Window^®^ dryer used in this work.

The strawberry puree was dried using the prototype shown in Figure 1 to reproduce the Refractance Window^®^ principle. For this purpose, samples were placed on a polyethylene membrane transparent to the passage of infrared energy (Mylar^®^_,_ 0.4 mm thick, Mylar Specialty Films, Hopewell, VA, USA), which remained in contact with the surface of the water contained in the drying prototype. The process was carried out in two stages. First, the fresh puree was dried at 50 °C for 90 min to reduce the initial moisture content. Subsequently, free cells (FC) or microcapsules (A-C, A-WPC, P-C, or P-WPC) were added independently to the partially dried puree. These samples were then subjected to a second drying step at 40 °C, 45 °C, or 50 °C. For this, samples were placed on the polyethylene membrane using a 2 mm thick template with circular shapes of 20 mm diameter. The puree was then spread over this template, filling the holes to form disks of strawberry puree.

There were two (2) control treatments for drying: strawberry puree without probiotics (PWP) and puree with unencapsulated free cells (PFC). Similarly, PAC corresponded to the puree with alginate–chitosan (A-C) capsules; PAWPC was assigned to the treatment of puree with alginate–whey protein concentrate (A-WPC) capsules; PPC was used for the treatment of puree with pectin–chitosan (P-C) capsules; and finally, PPWPC corresponded to the treatment of puree with pectin–whey protein concentrate (P-WPC) capsules.

After the second drying stage, the viability of the probiotic bacteria (from 60 min of drying, with 15 min intervals), the *a_w_* values, and the drying kinetics (from 0 min, in 15 min intervals, until the time at which samples had viability ≥10^6^ CFU/g) were evaluated.

### 2.12. Viable Microencapsulated Bacteria in Snacks

For viability assessment, 0.5 g of dehydrated puree was diluted in 4.5 mL of sodium citrate solution, followed by shaking at 2000 rpm for 5 min. Subsequently, 0.1 mL of serial dilutions were plated on MRS agar and incubated at 37 °C for 72 h.

Additionally, the presence of lactic acid bacteria was measured in fresh puree without the addition of *L. reuteri*. The result indicated that the strawberry puree contained <10^2^ CFU/g, which was undetectable after the first drying step.

### 2.13. Surviving Bacteria

The percentage of surviving bacteria, in both their free and encapsulated forms, was calculated using Equation (2), where *N* (CFU/g) was the number of viable probiotic bacteria after processing and *N*_0_ (CFU/g) corresponded to the initial bacterial count.

(2) $$Survivors\;\left(\%\right)=\left(\frac{N}{N_{0}}\right)\times 100\%$$

### 2.14. Design of Experiments and Statistical Analysis

Two experimental designs were used: one for microencapsulation and another for drying. In the case of microencapsulation, a 2^2^ factorial design was used, where the factors were the wall material (alginate and pectin) and the coating material (chitosan and whey protein concentrate). For this first experimental design, the response variables were viability and capsule size.

A completely randomized multilevel factorial design was used in the drying of the puree by the Refractance Window^®^ method, where the factors were the addition of microcapsules in the strawberry puree (A-C, A-P, P-C, and P-WPC) and the drying temperature (40 °C, 45 °C, and 50 °C). For this second experimental design, the response variables were the viability of the probiotic bacteria (taken from 60 min of drying, with 15 min intervals), the *a_w_* value, and the drying kinetics (from 0 min, in 15 min intervals, until the time at which samples had viability ≥10^6^ CFU/g) of the second drying stage.

The experiments were repeated three times. Analyses were performed in duplicate for each repetition (*n* = 3 × 2). Means and standard deviations were calculated for all data. Analysis of variance (ANOVA) was performed to determine the effect of process variables on response variables. Significant differences between means were determined at a 95% confidence level (*p* ≤ 0.05). To identify which treatments were significantly different, a Tukey comparison test was applied. The statistical program Infostat version 2020I was used.

## 3. Results and Discussion

### 3.1. Microcapsule Formation

The *L. reuteri* DSM 17938 concentration in the alginate and pectin solutions for the stage of microencapsulation was 10^10^ CFU/g. Upon atomization of solutions in calcium chloride, capsules were formed due to the ionic reaction between the COO^−^ (carboxylate) groups of the alginate and the divalent cations of calcium chloride, Ca^2+^. This reaction resulted in the formation of a gel network [24,25]. Similarly, capsule formation occurred with the pectin solution because this polysaccharide, with a low degree of esterification as used in this work, was able to form stable gels through ionotropic crosslinking in the presence of Ca^2+^ ions [26].

### 3.2. Capsules with Polymeric Coating

**Figure 2:**
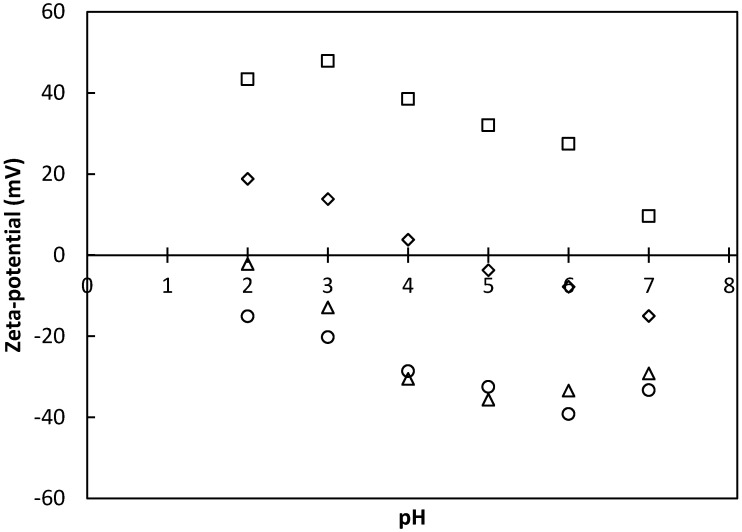
Z-potential as a function of pH for alginate (○), pectin (∆), chitosan (□), and whey protein concentrate (◊).

To coat the alginate or pectin capsules, chitosan or whey protein concentrate was used independently. This coating process was possible because during ionic gelation, not all of carboxylic groups of the pectin or alginate interacted with calcium ions, allowing an excess of negative charges on the surface of gel particles. This, in turn, allowed them to interact with an oppositely charged polyelectrolyte to form a protective layer on the capsule surface [27]. These interactions between oppositely charged polyelectrolytes can occur in polysaccharide–polysaccharide and/or polysaccharide–protein complexes [28]. According to Ye [29], this depends on factors such as pH. On this topic, Figure 2 shows the Z-potential values of materials used to form capsules in this work as a function of pH.

According to de Vos *et al.* [27] the Z-potential is a measure of the surface electrical charge and can predict interfacial reactions between a biomaterial and the surrounding material. Figure 2 shows that at pH 2, pH 3, and pH 4, the whey protein concentrate had the opposite charge of pectin and alginate. However, it was observed in preliminary tests that at pH 2 and pH 3, the whey protein concentrate solution had phase separation, which hindered the coating of the capsule. This behavior did not occur at pH 4.0, and so it was determined that this was the most appropriate pH for coating, as also reported by Gebara *et al.* [8] when using whey protein concentrate during ionic gelation.

The chitosan solution was used at pH 3.0, since, at this value, it showed the opposite charge of both pectin and alginate (see Figure 2). Similar analyses regarding this parameter for the interaction between pectin and chitosan were reported by Li *et al.* [30], who indicated that the pH value in relation to the Z-potential was of great importance, since it provided information on ranges where the surface of the microsphere was found to be positively or negatively charged such that electrostatic interaction between components could occur.

### 3.3. Encapsulation Efficiency

During production of microcapsules, the viability of probiotic bacteria was kept in a range near 10^10^ CFU/g. The average encapsulation efficiency was 91%, which could be considered high encapsulation performance, attributable to the process conditions, mainly with the whey protein concentrate coating treatments, which were carried out at 25 °C without the use of organic solvents and with a pH of 4.0 to favor electrostatic interaction between molecules [8,31]. These high encapsulation efficiency values are critical because when encapsulates are introduced into foods, microparticles can provide a sufficiently high number of probiotics that survive the processing conditions and are consumed in adequate amounts [31]. Finally, the encapsulation efficiency results showed no significant differences between treatments (*p* > 0.05).

Comparison of encapsulation efficiency with other research is sometimes difficult due to the use of different microorganisms, techniques, and wall materials [8]. However, the encapsulation efficiency results of this work were high compared to some reported by other researchers. For example, Corbo *et al.* [32] found an encapsulation efficiency of 83% using alginate with ionic gelation for the encapsulation of *Lactobacillus delbrueckii* subsp. *bulgaricus*. Additionally, Gebara *et al*. [8] obtained encapsulation efficiency of 84% when encapsulating *L. acidophilus* with pectin and whey protein concentrate. Elsewhere, Raddatz *et al*. [33] and Raddatz *et al*. [31] reported encapsulation efficiency values of approximately 78% and 92%, respectively, in the encapsulation of *L. acidophilus* with pectin. Finally, Chávarri *et al*. [34] found encapsulation efficiency values ranging between 20% and 40% for the encapsulation of *Lactobacillus gasseri* and *Bifidobacterium bifidum*, also using ionic gelation with a chitosan coating. In contrast, De Prisco *et al*. [21] reported higher encapsulation efficiency (97%) of the same probiotic strain used in this study during microencapsulation using vibratory technology with alginate.

As evidenced in this study, the internal ionic gelation technique, along with the wall materials used, proved to be effective in ensuring the survival of microorganisms during the microencapsulation process, which was a promising result for the protection of probiotics and their subsequent application in food.

### 3.4. Microcapsule Size

The size of capsules did not differ significantly from one treatment to another (*p* > 0.05), ranging from 100 to 300 µm, 110 to 302 µm, 120 m to 290 µm, and 120 to 300 µm for alginate–chitosan (A-C), alginate–whey protein concentrate (A-WPC), pectin–chitosan (P-C), and pectin–whey protein concentrate (P-WPC) capsules, respectively. The above were slightly smaller sizes than those found in recent studies that performed an extrusion technique, also using calcium chloride and alginate [35,36,37], and were similar to those reported in investigations employing vibrational technology [21,38].

The choice of capsule size is of great importance for application in various foods [35]. Marques da Silva *et al*. [39] recommend capsule sizes between 100 μm and 200 μm for food applications, as this size range allows the addition of encapsulated probiotics to a wide variety of products without causing an uncomfortable mouthfeel. In this research, most capsules were in that size range, which was appropriate, given that the application targeted a fruit puree matrix.

### 3.5. Characterization of the Strawberry Puree

The fresh strawberry puree had a moisture content of 11.6 g water/g *db*, 7° Brix, an *a_w_* of 0.98, and a pH of 3.4. It is important to note that the pH of strawberry pulp should not be lower than 3.3 for product quality reasons. In fact, the pH value did not vary with drying in this work.

During the first drying stage (*i.e.,* 50 °C for 90 min) the moisture content of the strawberry puree decreased to (2.1 ± 0.1) g water/g *db*, and the *a_w_* decreased to 0.94. Before the second drying stage, free cells (FC) or microcapsule (A-C, A-WPC, P-C, or P-WPC) were added independently to the partially dried puree, thus obtaining strawberry purees PFC, PAC, PAWPC, PPC, or PPWPC without a second drying step. At this point, before starting the second drying stage, there were no statistical differences (*p* > 0.05) in the moisture, *a_w_*, or pH values among purees without a second drying step (*i.e.*, PWP, PFC, PAC, PAWPC, PPC, and PPWPC); these parameters being on average (2.5 ± 0.3) g water/g *db*, 0.96 ± 0.00, and 3.4 ± 0.0, respectively, for all purees.

The above values were similar to those reported by Raddatz *et al*. [31] in strawberry puree enriched with free and encapsulated probiotic bacteria.

### 3.6. Drying Kinetics and a_w_

The quantity of probiotic bacteria remained around 10^9^ CFU/g until just before starting the second drying stage, which was consistent with typical values found in fruits enriched with probiotic bacteria [40]. Since the intention was to preserve the maximal survival of *L. reuteri* DSM 17938 in the strawberry snack, it was essential to maintain both the viability of the bacteria and the quality of the fruit.

In this section, the effect of temperature and time on the moisture content of strawberry puree was evaluated using Refractance Window^®^ technology. Analyses of moisture content, *a_w_*, and bacterial viability were important for this evaluation. In addition, it is important to remember that for probiotic microorganisms to have a positive impact on consumer health, the final product must contain an amount ≥10^6^ CFU/g of final product [41]. For this reason, the drying process in this work was carried out until the water content was reduced as much as possible while maintaining a viability level of at least 10^6^ CFU/g.

**Figure 3:**
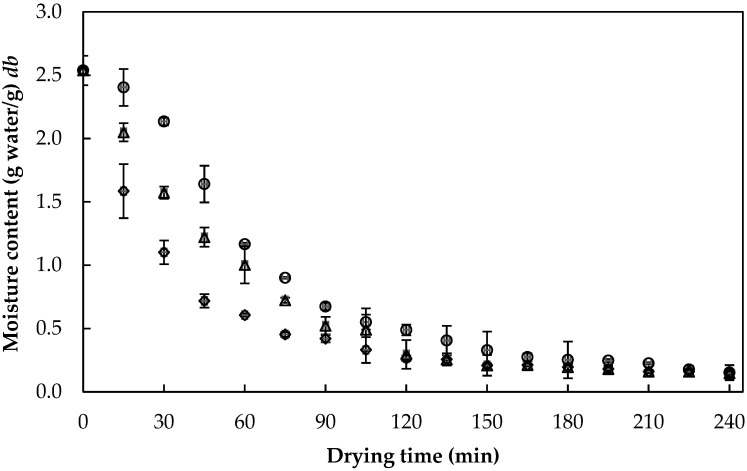
Drying curve of strawberry puree at 40 °C (○), 45 °C (∆), and 50 °C (◊).

The kinetic behavior had no statistically significant differences (*p* > 0.05) among snacks (PWP, PFC, PAC, PAWPC, PPC, and PPWPC), but it did significantly differ (*p* ≤ 0.05) among temperatures (40 °C, 45 °C, and 50 °C), where significant differences in moisture content and *a_w_* values were evident. Figure 3 shows representative kinetics of the drying process for strawberry puree at different temperatures.

It is evident in Figure 3 that, for the same drying time, the moisture content of the sample decreased significantly (*p* ≤ 0.05) as the temperature increased. This behavior resembled what was reported by Rodrigues *et al*. [42] when drying apple slices with hot air impregnated with probiotic microorganisms. In that study, authors indicated that higher temperatures increased both the mobility of water within the product and the energy available in the medium for water evaporation, thus facilitating the drying process. Such a trend could also be explained by the increased rate of heat transfer to particles at higher temperatures, which increased the driving force for moisture evaporation and, consequently, led to increased water removal from the food matrix [43].

Table 1 shows the moisture content and *a_w_* achieved after the drying process in strawberry snacks (PFC, PAC, PAWPC, PPC, and PPWPC) while maintaining a cell viability condition ≥10^6^ UFC/g. Drying should be an effective method to reduce *a_w_* < 0.6 for the production of desired non-perishable food products or food ingredients [44]. In that sense, as observed in Table 1, the Refractance Window^®^ drying process used in this work was effective in reducing the *a_w_* < 0.6 for only two snacks. These treatments were purees that were combined with alginate and pectin microcapsules, both coated with whey protein concentrate and dried at 40 °C for 240 min with Refractance Window^®^ technology (*i.e.*, PAWPC—40 °C and PPWPC—40 °C in Table 1, respectively). These two treatments prioritized the preservation of the viability of free cells (*i.e.*, ≥10^6^ CFU/g), using the lowest drying temperature (*i.e.*, 40 °C), which resulted in snacks with greater quality, as the low temperature would help preserve the nutritional quality of the puree [15]. This behavior could be explained by the membrane-stabilizing properties of whey protein concentrate, which can prevent cell damage [4]. In contrast, during preliminary tests, chitosan-coated capsules showed reduced viability, perhaps due to cracks in the chitosan coating of the treated capsules. Additionally, Niu *et al*. [45] stated that chitosan releases encapsulated substances upon possible pH changes, which may have occurred with the strawberry matrix in this study.

Also, within the safe limits of conservation (*a_w_* ≤ 0.6) were snacks produced from purees combined with alginate–chitosan microcapsules dried at 40 °C (PAC—40 °C) for 210 min and 45 °C (PAC—45 °C) for 135 min (see Table 1).

The high *a_w_* values at high temperatures in Table 1 were due to the higher moisture content in these snacks. Due to the need to maintain viability ≥10^6^ CFU/g, the drying time of these samples was considerably reduced, resulting in an insufficient decrease in their water content. In fact, the lowest water content values were also observed in snacks from purees that were combined with alginate or pectin microcapsules coated with whey protein concentrate and dried at 40 °C for 240 min (PAWPC—40 °C and PPWPC—40 °C in Table 1, respectively), with values of 15% moisture, which, according to authors, would guarantee the stability of these snacks during storage [46,47]. Additionally, low moisture content and *a_w_* are beneficial because they prevent the growth of undesirable microorganisms, lipid oxidation, and Maillard browning [48].

### 3.7. Viability during Drying

**Figure 4:**
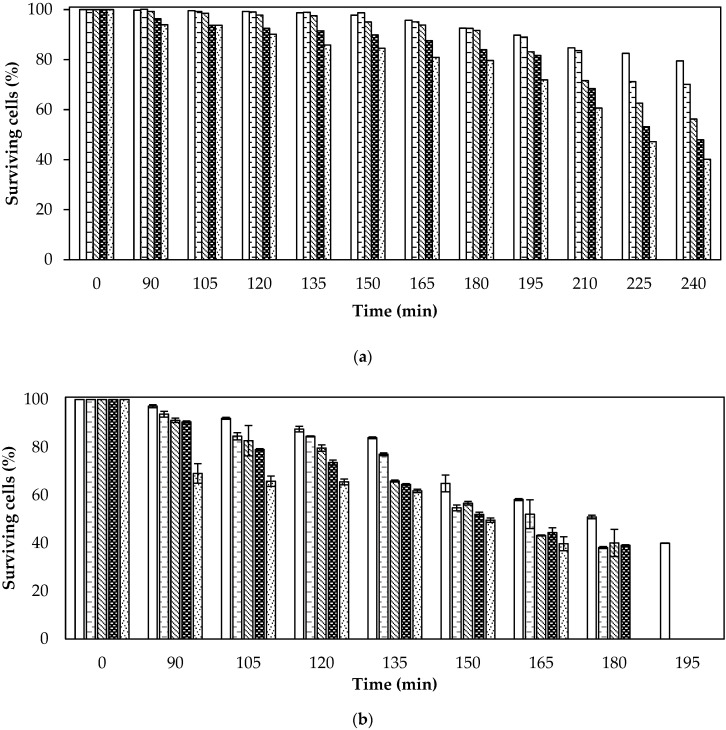

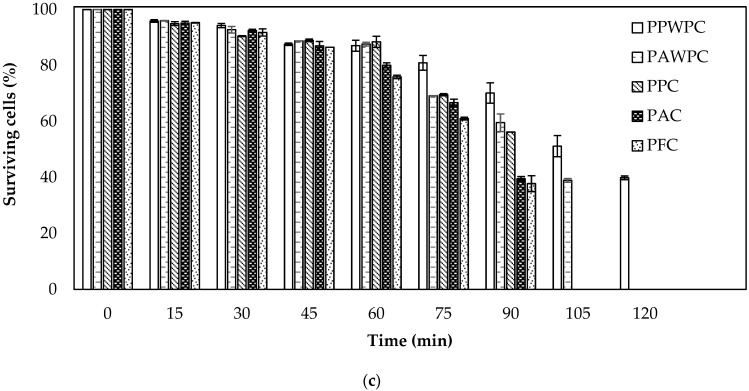
Cells surviving after Refractance Window^®^ drying as a function of time for purees with free cells (PFC), alginate–chitosan (PAC) microcapsules, alginate–whey protein concentrate (PAWPC) microcapsules, pectin–chitosan (PPC) microcapsules, and pectin–whey protein concentrate (PPWPC) microcapsules at (**a**) 40 °C, (**b**) 45 °C, and (**c**) 50 °C.

Figure 4 shows the percentages of cells surviving drying as a function of time for purees with free cells (PFC) and microcapsules of alginate–chitosan (PAC), alginate–whey protein concentrate (PAWPC), pectin–chitosan (PPC), and pectin–whey protein concentrate (PPWPC) at different drying temperatures using Refractance Window^®^ technology (40 °C, 45 °C, and 50 °C).

Figure 4 clearly shows that the lower the drying temperature, the lower the loss of viability for all treatments. In the case of the cell-free puree treatments dried at 45 °C and 50 °C, there were viable cells up to 165 min and 90 min, respectively, while this same treatment dried at 40 °C preserved cell viability throughout the drying time, although at 240 min the amount was reduced to 10^4^ CFU/g, which was below the threshold intended in the snacks in this work (*i.e.*, ≥10^6^ CFU/g). However, as shown in Figure 4, 40 °C proved to be the best temperature to preserve the viability of probiotic bacteria, a result in accordance with the claims of Zura-Bravo *et al*. [49], who suggested that this drying temperature was appropriate for drying fruits impregnated with bacteria, although they emphasized that the resistance of bacteria might vary according to their strain.

Figure 4 also reveals a decrease in the viability of probiotic bacteria as the temperature increased during the drying process, which could be related to several factors, such as protein denaturation, intracellular water elimination, and lipid oxidation, that alter the permeability of the cell membrane. Additionally, high temperature, low humidity, and decreased *a_w_* of the product could generate stress in bacteria [50]. Finally, the inactivation of cells in treatments with high *a_w_* values (see Table 1) could be attributed to the higher thermal conductivity of water molecules, which may have transferred heat more efficiently to cells in the puree [51].

According to Figure 4, encapsulation provided additional protection to probiotic bacteria, as it allowed their viability to be maintained for a longer time during the drying process, compared to the puree treatment with free cells added; this was true at all three of the drying temperatures studied. However, even with encapsulation, temperature was a determining factor in the viability of the probiotics.

It is further highlighted in Figure 4 that prolonged processing times negatively affected the viability of probiotic cells. The above is true because once the water had evaporated from the matrix, the temperature of the dried particles gradually increased, which promoted progressive thermal damage [51,52]. Therefore, it is important to explore alternatives that reduce the exposure time of bacteria to the drying process, such as a previous decrease in moisture content. The above behavior has also been previously reported by other authors [53,54,55].

As previously mentioned, purees that were combined with alginate or pectin microcapsules coated with whey protein concentrate and dried at 40 °C (PAWPC—40 °C and PPWPC—40 °C in Table 1, respectively) were the most effective in terms of moisture content and *a_w_*, important factors for obtaining a dehydrated strawberry puree as desired in this work. These treatments maintained viabilities of 10^7^ CFU/g and 10^8^ CFU/g, respectively. This exceptional performance of the PAWPC—40 °C and PPWPC—40 °C treatments (see Table 1 and Figure 4) in terms of probiotic viability could be attributed to several reasons. For example, both treatments used polymeric whey protein concentrate coating, whose components, such as α-lactalbumin and β-lactoglobulin, have been shown to confer thermal resistance when used as capsule coatings, thus providing enhanced protection against heat [56]. Alternatively, the strong interaction of whey protein concentrates with alginate and pectin at pH 4.0 could have reduced oxygen permeability. Additionally, whey protein concentrate could be a source of nutrients for probiotic bacteria [57].

As for chitosan-coated capsules, the decrease in the viability of probiotic bacteria may have been due to the presence of cracks. Although not all capsules may have exhibited these cracks, it is possible that a proportion of probiotic bacteria may have been exposed to adverse environmental conditions during drying. Chitosan, recognized for its antimicrobial activity and antifungal properties, would also explain the reduced survival of bacteria encapsulated with this material [58]. However, it is important to note that chitosan plays an important role in protecting bioactive compounds, such as probiotic bacteria, against pathogens present in the environment.

According to Kong *et al*. [59] and Baptista *et al*. [60] the decrease in bacterial counts in systems with chitosan was due to the interaction of positively charged amino groups with polyanionic components of cell walls, leading to changes in cell permeability and loss of cellular compounds. Meanwhile, Yonekura *et al*. [61] found similar behavior to what was observed in this study when encapsulating *L. acidophilus* NCIMB 701748 in alginate and chitosan materials, reporting that the loss of viability was due to the interaction between chitosan and cell walls, in addition to the stress caused by the encapsulation technique. Likewise, Vanden Braber *et al*. [62] pointed out that chitosan, due to its natural antimicrobial capacity, could result in a decrease in bacterial counts by forming a polymeric film around the bacterial surface, blocking the passage of nutrients and oxygen, which led to the death of microorganisms.

Based on the above, a strong influence of the coating material on the viability of *L. reuteri* encapsulated and incorporated in strawberry puree and subjected to Refractance Window^®^ drying processes was evidenced.

## 4. Conclusions

The application of the ionic gelation technique followed by polymeric coating proved to be highly efficient in preserving the survival of *Lactobacillus reuteri* DSM 17938 during the production of the proposed strawberry snack by Refractance Window^®^ drying.

Even under adverse processing conditions, the presence of protective barriers made it possible to maintain the viability of bacteria in the recommended ranges (*i.e.,* ≥10^6^ UFC/g final product). Additionally, the methodology used allowed the formation of capsules with a suitable particle size (*i.e.,* 200 µm) for application in the food industry.

It was evidenced that the drying temperature exerted a significant impact on the viability of both free and encapsulated probiotics in the strawberry matrix. Importantly, microencapsulation of *L. reuteri* DSM 17938 in alginate and pectin with a whey protein concentrate coating was able to maintain bacterial survival in the strawberry puree during the Refractance Window^®^ drying process at 40 °C. These treatments showed promise for obtaining dehydrated strawberry snacks with probiotic viability, as they resulted in optimal moisture content for a functional food.

This study provides a promising methodology for the production of strawberry snacks in which the viability of bacterial strains is maintained even under drying processing conditions. These results could be critical for the food industry, as they could enable the development of products that could not only be appealing to the senses but also offer significant probiotic health benefits to consumers.

Limitations of this study include potential variations in raw materials and the need for further exploration of scale-up challenges. Future research should address these issues, standardize raw materials, and focus on optimizing large-scale production. The release kinetics of the probiotic from the microcapsules in the manufactured snack should also be evaluated. Additionally, investigating alternative encapsulation materials and assessing the impact of different fruit matrices on probiotic viability would enhance the methodology’s robustness and applicability in diverse food products.

## Figures and Tables

**Table 1:** Moisture content and water activity (*a_w_*) retaining viability condition ≥10^6^ CFU/g.

Treatment	Time (min)	g Water/g *db*	*a_w_*
PFC—40 °C	225	0.25 ± 0.00 ^ab^	0.69 ± 0.00 ^cd^
PAC—40 °C	210	0.20 ± 0.02 ^ab^	0.58 ± 0.07 ^ab^
PAWPC—40 °C	240	0.15 ± 0.01 ^a^	0.52 ± 0.02 ^a^
PPC—40 °C	225	0.21 ± 0.01 ^ab^	0.64 ± 0.00 ^bc^
PPWPC—40 °C	240	0.15 ± 0.00 ^a^	0.54 ± 0.01 ^ab^
PFC—45 °C	135	0.25 ± 0.01 ^ab^	0.70 ± 0.00 ^cd^
PAC—45 °C	135	0.25 ± 0.02 ^ab^	0.58 ± 0.01 ^ab^
PAWPC—45 °C	135	0.26 ± 0.05 ^ab^	0.69 ± 0.04 ^cd^
PPC—45 °C	135	0.28 ± 0.01 ^b^	0.72 ± 0.00 ^cde^
PPWPC—45 °C	150	0.26 ± 0.08 ^ab^	0.70 ± 0.01 ^cd^
PFC—50 °C	75	0.43 ± 0.02 ^cd^	0.91 ± 0.01 ^g^
PAC—50 °C	60	0.54 ± 0.03 ^d^	0.81 ± 0.03 ^efg^
PAWPC—50 °C	75	0.45 ± 0.01 ^cd^	0.79 ± 0.00 ^def^
PPC—50 °C	75	0.49 ± 0.01 ^cd^	0.84 ± 0.01 ^fg^
PPWPC—50 °C	90	0.42 ± 0.02 ^c^	0.85 ± 0.04 ^fg^

Puree with free cells (PFC), alginate–chitosan microcapsules (PAC), alginate–whey protein concentrate microcapsules (PAWPC), pectin–chitosan microcapsules (PPC), and pectin–whey protein concentrate microcapsules (PPWPC); Means with a common letter (a, b, c, d, e, f, g) are statistically similar (*p* ≤ 0.05).

## Data Availability

The data that support the findings of this study are available from the corresponding author upon reasonable request. The data is designed to be used in other ongoing research and should be protected before official publication.
